# The Distribution and Common Amino Acid Polymorphisms of Human Papillomavirus (HPV)-31 Variants in 2700 Women from Northern China

**DOI:** 10.1371/journal.pone.0099141

**Published:** 2014-06-05

**Authors:** Mengfei Liu, Zhonghu He, Longfu Xi, Jingjing Li, Fangfang Liu, Ying Liu, Yaqi Pan, Tao Ning, Chuanhai Guo, Ruiping Xu, Lixin Zhang, Hong Cai, Yang Ke

**Affiliations:** 1 Laboratory of Genetics, Key laboratory of Carcinogenesis and Translational Research (Ministry of Education), Peking University Cancer Hospital & Institute, Beijing, P. R. China; 2 Department of Pathology, School of Medicine, University of Washington, Seattle, Washington, United States of America; 3 Anyang Cancer Hospital, Anyang, Henan, P. R. China; National Institute of Health - National Cancer Institute, United States of America

## Abstract

To investigate the distribution of Human papillomavirus (HPV)-31 A, B and C variants as well as the common amino acid polymorphisms in Chinese women, all 14 HPV-31 positive cervical exfoliated cell specimens identified from a descriptive study including ∼2700 women from Northern China were analyzed. HPV-31 positive specimens were identified by Mass Spectrometry and the fragments of partial Long Control Region, E6 and E7 were amplified and directly sequenced or cloned into vector and then sequenced to confirm the variant information. HPV-31 prevalence in Northern Chinese female population was 0.52%. Six different sequences represented all 14 isolates, and these isolates were subsequently classified into variant lineage A (9), B (0) and C (5) by phylogenetic analysis. Five common amino acid polymorphism sites (2 in E6 and 3 in E7) and a novel non-synonymous mutation were detected in the current study. Our investigation suggested that HPV-31 was much less detected in Chinese women population than that in western countries. A and C variants were commonly detected while B variants were rarely detected in this population.

## Introduction

Human papillomavirus (HPV) is a family of double strand DNA viruses. The classification of HPV is based on the conserved L1 sequence coding for the major capsid protein [1]. HPV can be classified as types, subtypes and variants when the differences between their L1 sequences are more than 10%, 10%–2% and less than 2%, respectively [1]. To date, more than 150 types of HPV have been identified [2]. According to the carcinogenicity in cervical cancer, HPVs are classified as high risk and low risk types [3]. Persistent infection of high risk types has been proven to be a necessary cause of cervical cancer and risk factor of some other epithelial-derived carcinomas, such as ano-genital and oropharyngeal cancer etc. [4]. HPV-16 and HPV-18, as the most prevalent high risk HPV types, account for 70% cervical cancer cases worldwide [3]. In addition, it has been reported that the potentiality of carcinogenesis is different among HPV variants. Results of longitudinal study show that the non-European variants of HPV-16 and HPV-18 are significantly more likely to cause persistent infection and CIN3+ than European variants [5]. Non-synonymous mutations in the E6, E7 oncogenes in HPV genome may alter the biological or immunogenic properties of the encoded protein, causing discrepancies in carcinogenicity of variants [6].

HPV-31 is one of the high risk types most closely related to HPV-16. To date, studies have shown many intratypic variations in the region of L1, E6, E7 and Long Control Region (LCR) of HPV-31 worldwide [7]–[13]. Data of whole-genome sequencing study suggests that HPV-31 can be phylogenetically classified as A, B and C variant lineages according to the nucleotide sequences of isolates [14]. Xi et al. in the US have drawn the discrepancies of A, B and C variants in their viral properties from epidemiological studies. The clearance of A variants were significantly faster than C variants among African-American women (*P* = 0.05), but this trend was not observed in Caucasian women (*P* = 0.85) [15]. Also, the odds ratio of CIN2/3 was 1.7 (95% CI: 1.0–2.9) for infections with A variants and 2.2 (95% CI: 1.2–3.9) for infections with B variants as compared to those with C variants [16]. Moreover, they found in the US female population, proportions of A, B, C variants of HPV-31 in Caucasian women were 48.7%, 17.8%,33.5%, and 22.9%, 17.4%, 59.6% in African-American women (*P*<0.001), respectively [16].

To date, studies on cervical infection of HPV-31 variants have mainly been conducted in western countries, and little information has been provided in Asian countries, especially China. In this study, cervical exfoliated cell specimens collected from 2700 women in Northern China were tested, and the distribution of HPV-31 variant lineages and amino acid polymorphism was described.

## Material And Methods

### Study subjects

In 2007–2009, a population-based esophageal cancer cohort study was initiated in 9 villages in rural Anyang, China [17], and the eligibility criteria were as follows: 1) permanent residency in the target villages; 2) aged 25–65 years; and 3) no history of cancer, cardiovascular disease or mental disorder. During 2009 to 2010, cervical exfoliated cells of 2703 female cohort members from 7 out of 9 target villages were collected for the detection of HPV DNA.

### Ethics statement

Research protocols and materials of this study were approved by the Institutional Review Board of the Peking University School of Oncology, China. All participants in this study provided written informed consent.

### Specimen and data collection

Exfoliated cells from the cervix were collected by experienced gynecologists using saline-soaked swabs and cells were rinsed into an eppendorf tube filled with saline. All specimens were then centrifuged at 5000 rpm for 5 min and supernatants were discarded. All specimens were stored at −20°C and subsequently transported to our laboratory in Beijing and stored in ultralow temperature freezers (−70°C) until the DNA extraction and HPV detection.

A questionnaire was completed by all participants during the interview to obtain the demographic data and personal information including age, place of residence, tobacco use history, together with the number of sexual partner and pregnancy history etc.

### Identification of HPV-31 variant

DNA was extracted using Biomek 3000 system (Beckman Coulter, Brea, CA, USA) and then tested using Mass Spectrometry (MS) to detect infections of HPV 68, 66, 59, 58, 56, 52, 51, 45, 39, 35, 33, 31, 18, 16, 11 and 6 (BGI, Shenzhen, China) [18]. All HPV-31 positive specimens were subsequently subjected to the variant analysis. A pair of external primers was used to amplify an ∼1000 bp fragment from nucleotide position 7810 to 897 of the HPV-31 covering 3′ part of LCR, and the entire E6 and E7 regions, and Polymerase Chain Reaction (PCR) product was sequenced using one pair of external primers and one pair of internal primers [16]. PCR products were cloned into P-Easy Blunt vector (TransGen Biotech, Beijing, China) if the concentration of products was too low to perform the direct sequencing. Four colonies were picked and sequenced for each PCR product. The primer sequences were as following. The forward external primer: 5′-TGTTTAAACTGCCAAGGTTGTG, the reverse external primer: 5′-CATAAAACCAACCATTGCATCC, the forward internal primer: 5′-TGGAACAACATTAGAAAAATTGACA, the reverse internal primer: 5′-TCTTCTGGACACAACGGTCTTT. PCR was performed using Phusion High Fidelity Taq Polymerase (New England Biolabs, USA). The kit ABI PRISM BigDye Terminator Cycle Sequencing v 3.1 was used in the sequencing procedure. Sequences obtained were checked for quality using chromas software. Blast tool of NCBI was used to identify novel sequences. A viral isolate was defined as a novel variant if as compared with other isolates, there was one or more nucleotide variations in the region analyzed [1].

Duplicated PCR was performed in order to investigate the reproducibility of our method. A hundred percent identical sequences were observed from duplicated PCRs (data not shown).

### Statistical analysis

Multiple sequence alignments of the partial LCR, E6 and E7 ORFs and distance matrix bootstrap analyses were performed using Clustal-X program [19], the phylogenetic tree of HPV-31 variants was displayed using Tree View X [20]. Numbers on the nodes indicated the bootstrap values (1000 replicates) of branches. 95%CI of HPV-31 prevalence was calculated based on Poisson distribution.

## Results

Among the 2703 specimens, 14 (0.52%, 95%CI: 0.28%–0.87%) were HPV-31 positive (Table 1). Analysis of HPV-31 variant lineage distribution (N = 14) showed that, in China, A variants were the most commonly detected (64.3%), followed by C variants (35.7%), and no B variant was found (0%).

**Table 1:** HPV-31 detection rate by Mass Spectrometry, demographic characteristics, behavior variables and proportion of positive specimens stratified by variant lineages in women from Northern China.

Variables	Specimens from Northern China
MS Detection	
Specimens tested	2703
HPV-31 positive	14
HPV-31 detection rate (%) and 95%CI*	0.52(0.28–0.87)
Education level	
Below junior middle school (%)	9 (64.3)
Junior middle school or above (%)	5 (35.7)
Age distribution	
Median (years)	46
Interquartile Range (years)	40–56
Number of sexual partners	
N< = 1 (%)	14 (100)
N>1 (%)	0
Number of pregnancies	
< = 3 (%)	8 (57.1)
>3 (%)	6 (42.9)
Contraceptive usage	
Yes (%)	1 (7.1)
No (%)	13 (92.9)
Cigarette smoking	
Yes (%)	1 (7.1)
No (%)	13 (92.9)
Variant Lineage (N = 14)	
A variants (%)	9 (64.3)
B variants (%)	0 (0.0)
C variants (%)	5 (35.7)

*95% CI was calculated using Poisson distribution.

**Figure 1:**
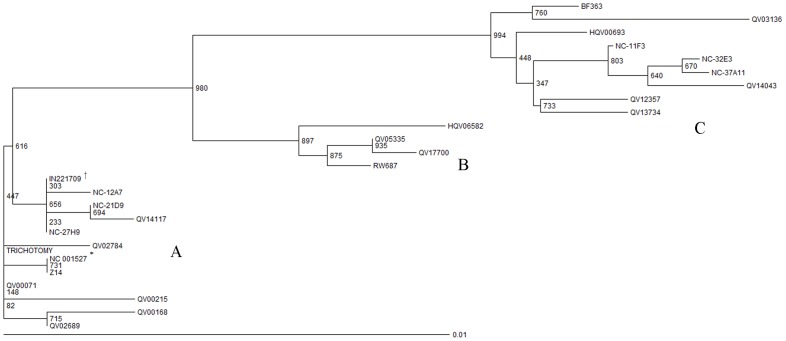
The Neighborhood Joining phylogenetic tree of HPV-31 variants. The tree was constructed using HPV-31 variants sequences of partial Long Control Region, E6 and E7 identified in Northern China (NC specimens, N = 6) and variants previously reported in whole genome study (N = 19) [14]. Numbers on the nodes indicated the bootstrap values (1000 replicates) of branches. ^*^ NC_001527 was the reference sequence of HPV-31 and it was not one of the sequences identified in the current study. ^†^ The sequence of NC-27H9 was the same as IN221709.

Five novel isolates were identified from 14 HPV-31 positive specimens (the sequence of NC-27H9 is identical with IN221709). Phylogenetic analysis based on Neighborhood Joining method was performed including all unique isolates in the current study (N = 6), accompanied by 19 sequences which had been reported in whole genome study [14]. Three distinct brunches were formed (Figure 1). Three isolates in the current study (NC-32E3, NC-11F3, and NC-37A11) were classified into C variant lineage; the other three isolates (NC-27H9, NC-21D9, NC-12A7) were classified into A variant lineage and no isolate was classified into B variant lineage.

Comparing with the prototype isolate, seven nucleotide alternations in the 3′ part of LCR,seven in E6, and six in E7 were identified and listed in Table 2. Five previously identified non-synonymous nucleotide alternations C285T (5/14), C520T (5/14), C626T (9/14), G695A (5/14) and A743G (14/14) were frequently detected (>25%) [7], [13], leading to amino acid changes H60Y, A138V in E6 protein of C variants, H23Y in E7 protein of A variants, E46K in E7 protein of C variants, and K62E in E7 protein of both A and C variants identified in China, respectively. In addition, one novel nucleotide alternation (A837G) was identified, which leads amino acid change from Asparagine to Serine at position 93 of E7 protein.

**Table 2:** Frequency and Genetic variability of cervical exfoliate cell derived HPV-31 variants in Long Control Region, E6 and E7 in Northern China.

		Long control region							Nucleotide position at E6							Nucleotide position at E7						
Variant name	Frequency (N)	7860	7880	17	18	37	45	87	176	285	320	326	404	428	520	580	626	670	695	743	837	Variant lineage
Ref*	-	G	T	A	G	G	G	T	C	C	A	A	G	A	C	G	C	C	G	A	A	A
NC-32E3	1	A	C	G	T	A	T	G	-	**T** a	T	G	A	G	**T** b	A	-	T	**A** d	**G** e	-	C
NC-37A11	1	C	C	G	T	A	T	G	-	**T** a	T	G	A	G	**T** b	A	-	T	**A** d	**G** e	-	C
NC-11F3	3	A	C	G	T	A	T	-	-	**T** a	T	-	A	G	**T** b	A	-	T	**A** d	**G** e	-	C
NC-21D9	3	-	-	-	-	-	-	-	T	-	-	-	-	-	-	-	**T** c	-	-	**G** e	-	A
NC-12A7	1	-	-	-	-	-	-	-	-	-	-	-	-	-	-	-	**T** c	-	-	**G** e	**G** f	A
NC-27H9†	5	-	-	-	-	-	-	-	-	-	-	-	-	-	-	-	**T** c	-	-	**G** e	-	A
Total	14																					

Letters in bold represented amino acid changes compared to the reference sequence.

*Reference sequence of HPV-31 was NC_001527 and this sequence was not one of the sequences identified in the current study.

a The H60Y amino acid polymorphism in E6.

b The A138V amino acid polymorphism in E6.

c The H23Y amino acid polymorphism in E7.

d The E46K amino acid polymorphism in E7.

e The K62E amino acid polymorphism in E7.

f The N93S amino acid polymorphism in E7.

† The sequence of NC-27H9 was the same as IN221709.

## Discussion

In this descriptive study, we found that the prevalence of HPV-31 in northern Chinese women was 0.52% (95%CI: 0.28%–0.87%). HPV-31 variants in our sample could be classified into 2 clades, representing A and C variant lineages. The A variants were the most commonly detected, followed by C variants, and no B variant was detected. Five identified (2 in E6 and 3 in E7) amino acid polymorphisms and one novel amino acid polymorphism in E7 were found in this population. To our knowledge, this was the first study focusing on HPV-31 variants in Chinese women and the population-based specimens gained representation for general population as compared to hospital-based studies.

Race-specific preferences of variant infection and discrepancies in viral properties have been identified and illustrated systematically in HPV-16. Phylogenetic analysis concerning viral sequences revealed that variants segregate based on the geographical origin and can be classified as European, African, Asian, Asian-American and North American [21], [22]. Epidemiological studies showed HPV-16 variants (e.g., D25E and L83V in E6) differ in their association with viral persistence, high grade lesions in cervix, and cervical cancer in various populations [23], [24]. And most importantly, evidences from functional and mechanistic studies indicated amino acid polymorphisms in E6 and E7 protein can affect the viral properties and the carcinogenicity of HPV variants. Such as: (1) ability to abrogate serum/calcium-dependent keratinocyte differentiation and to induce the in vitro degradation of p53 [25]; (2) immunogenicity of specific viral epitopes, resulting in induction of low neutralizing antibody titers or altered cell mediated response [26].

For HPV-31 variants, phylogenetic analysis did not show any racial or geographic clustering worldwide. This phenomenon was also observed in HPV-33, 35, 52, 58 and 67, which is different from that in HPV-16 and 18 [14]. One possible inference for the lack of association could be evolution mechanisms in HPV other than the virus-host co-evolution theory [21], for the fact that geographic, sexual, and racial separation doesn't significantly affect their viral sequences' evolution. However, viral-host interaction might still play an important role to explain discrepancies of viral properties among variant lineages in different populations.

In the current study, the HPV-31 prevalence observed was 0.52%, which was similar to the prevalence (0.4%) reported by meta-analysis in women with normal cytology from the Asia region [27]. Though the prevalence of HPV-31 is high in women with abnormal cervical cytology worldwide [28], the prevalence estimates vary geographically in women with normal cytology. We noticed that our prevalence of HPV-31 was much lower than that reported in Xi's study (1.1%) which enrolled 5060 women from the ASC-US and LSIL Triage study (ALTS) in US [16]. And this was in stand with an international study which reported that prevalence of HPV-31 in Asian continent (0.3%) was lower than the world average level (0.8%), especially lower than the Europe (2.3%) and Latin American (1.2%) in women with normal cytological findings [29]. Though mechanisms underlying the geographic variation of HPV-31 prevalence are largely unclear, ethnic difference among continents might play a role.

The current investigation only found variant lineage A (64.3%) and C (35.7%) in 2703 Chinese women which was notably different from Xi's study which reported the proportion of A, B and C variants were 41.7%, 21.1% and 37.2% in US female population [16]. We were aware that the relatively low prevalence of HPV-31 in our population led to the small number of positive specimens (14 out of 2700 women), which might in turn impair the statistical power of the reported proportions of variants and the comparison to US population as well. Fisher's exact test was performed using data in the current study added by results of previous population based study in Caucasian women and African-American women by Xi et al [16]. The distribution of HPV-31 variants in Chinese female population was significantly different from that in the African-American women (*P* = 0.004). However, the difference was not significant between Chinese women and Caucasian women (*P* = 0.221). This was an evidence to support the potential racial and geographic variation in HPV-31 variants distribution though phylogenetic analysis of HPV-31 isolates did not reveal ethnic clustering as observed previously for HPV-16 and 18. Besides Xi's study, another two hospital-based studies have also investigated cervical HPV-31 infections at the variant level. Chagas et al in brazil enrolled 35 HPV-31 positive specimens from women with abnormal cytology, and reported the proportion of A, B and C variants were 57.2%, 5.7% and 37.1%,respectively [7]. Ferenczi et al collected 41 HPV-31 positive specimens in Italy from women undergoing colposcopical examination for malignant or premalignant disease, and found the proportion of A, B and C variants were 4.9%, 29.3% and 65.8%, respectively [8]. But results from hospital-based studies have limited representation, for the presence of lesions may confound the real distribution of variants in general population. And this brings the uniqueness of the current study to examine variants in a relatively homogenous population (all Chinese subjects of Han race), with comparable socioeconomic conditions, at a geographic region of low HPV-31 prevalence.

Although epidemiological studies have shown the discrepancies of viral properties among HPV-31 variant lineages, it is still crucial to identify amino acid polymorphisms accounting for the difference, especially in E6 and E7 regions due to these oncoproteins are essential for the occurrence, development and maintenance of malignancy in cervical epithelial cells [30], [31]. In the current study, five common and one novel non-synonymous amino acid polymorphisms located in HPV-31 E6 and E7 genes (H60Y and A138V in E6; H23Y, E46K, K62E and N93S in E7) in Chinese women were found among HPV-31 lineages. Since the mechanisms causing different viral properties among HPV-31 variants are largely unknown, functional prediction may only be made from the corresponding amino acid sequence of HPV-16 E6 and E7 [31]–[35]. The H60Y locates after the putative p53 degradation site containing the amino acids FAF located at positions 45–47 on E6 [36], and the A138V locates after the putative site (amino acid 123) for p53 binding and degradation [36], [37]. The H23Y locates in the Rb binding domain [35], and the E46K locates between the Rb binding domain and zinc finger motif of E7 protein [35], [38]. Since E7 acts as the major immortalizing protein through Rb/E2F pathway, amino acid alternations in Rb binding domain may result in the change of E7's ability to immortalize cells among variants. The K62E in E7 have been identified repeatedly with an extremely high percentage [7], [13], which suggests most HPV-31 variants share this polymorphism, except for the prototype. In addition, two novel nucleotide alternations at position 87 and 837 were firstly reported. And only the A837G is a non-synonymous mutation, which leads to a N93S amino acid substitution. Further functional studies are necessary to identify the potential role of these amino acid polymorphisms in the discrepancy of viral persistence and risk of tumor progression among HPV-31 variant lineages.

In this descriptive study, we found that the prevalence of HPV-31 in Northern Chinese women was 0.52% (95%CI: 0.28%–0.87%). The variant lineage A (9/14) of HPV-31 was the most commonly detected variants, followed by C variants (5/14), which was notably different from those in some western ethnic populations. Five frequent non-synonymous mutations among HPV-31 variant lineages in E6 and E7 protein (H60Y and A138V in E6; H23Y, E46K and K62E in E7) and a novel non-synonymous mutation (N93S in E7) were detected. This study provides important basic data for the subsequent association studies and related molecular mechanism studies on HPV-31 variants and cervical lesions in Chinese women.
